# A Synthesized Model for Applying Stress Management and Biofeedback Interventions in Research Utilization: A Systematic Review and Meta-analysis

**DOI:** 10.2174/0117450179276691231229071003

**Published:** 2024-02-28

**Authors:** Manyat Ruchiwit, Sararud Vuthiarpa, Kampol Ruchiwit, Kasorn Muijeen, Kanjanee Phanphairoj

**Affiliations:** 1 Faculty of Nursing, Rattana Bundit University, Pathumthani, Thailand; 2 Faculty of Allied Health Sciences, Thammasat University, Pathumthani, Thailand; 3 Faculty of Nursing, Thammasat University, Pathumthani, Thailand; 4 Faculty of Nursing, Chulalongkorn University, Bangkok, Thailand

**Keywords:** Stress management, Biofeedback interventions, Research utilization, Systematic review, Meta-analysis, Mental health

## Abstract

**Background:**

Stress management and biofeedback interventions have been shown to be effective in improving mental and physical health outcomes. However, previous research studies and synthesized models for applying these interventions in research utilization are insufficient.

**Objective:**

This study aimed to synthesize a model for applying stress management and biofeedback interventions in research utilization.

**Methods:**

A systematic review and meta-analysis were conducted according to the PRISMA guidelines.

Multiple studies were used to assess the effectiveness of applying stress management and biofeedback interventions published from 2017 to 2023. The process included identifying the research questions, conducting a comprehensive literature search, assessing study quality, extracting data, synthesizing the data, analyzing and interpreting the findings, drawing conclusions, and making recommendations.

**Results:**

The results indicated a significant mean effect size without evidence of publication bias. The effect sizes of the subgroups among the study variables were not significantly different [*Q* = 4.02, *p* = .26]. However, there were significant differences regarding the mean effect sizes among the studies [*Q* = 63.59, *p <* .001] and also in terms of the test of subgroups among the participants [*Q* = 8.49, *p =* .04].

**Conclusion:**

The results emphasize the importance of evidence-based practice and highlight the need for ongoing evaluation and refinement of interventions. The proposed model was supported by related theories and research studies in order to ensure the robustness and reliability to guide practice and future research in the field of biofeedback interventions. By following this model, researchers and practitioners can ensure that stress management and biofeedback interventions are evidence-based and are effective in improving mental and physical health outcomes.

## 1 INTRODUCTION

Stress management is the process of utilizing techniques and interventions to reduce the negative effects of stress on the body and mind [1-5]. There are various approaches to stress management, including relaxation techniques [6-9]. These approaches aim to reduce the physiological response to stress and promote relaxation [10]. Biofeedback, an intervention used in stress management, involves monitoring physiological responses, such as heart rate, skin temperature, brain wave, and muscle tension, providing feedback to help individuals learn to control their responses [11-13]. Biofeedback interventions have been shown to be effective in reducing stress and promoting relaxation among individuals in various settings, such as healthcare, education, and the workplace [14-17]. While stress management and biofeedback interventions have been shown to be effective [5, 18-22], and despite the growing evidence related to the effectiveness of these interventions, previous research studies and synthesized models for applying these interventions in research utilization are inadequate and ambiguous, and their application in nursing practice remains suboptimal.

In light of this gap, the objective of this study is to synthesize a comprehensive model for the application of stress management and biofeedback interventions in research utilization. By conducting a systematic review and meta-analysis of the existing literature, this study aims to consolidate the available evidence and develop an evidence-based framework that can guide the integration of these interventions into research practices. The synthesized model provides a clear and practical roadmap for researchers, healthcare professionals, and practitioners to effectively implement stress management and biofeedback interventions within their respective fields. Through the synthesis of existing knowledge and the development of this model, it is anticipated that the utilization and impact of stress management and biofeedback interventions in research can be significantly enhanced, ultimately leading to improved outcomes for individuals experiencing stress-related challenges.

### 1.1 Research Questions

1. What is the effectiveness of biofeedback interven-tions in reducing stress and improving health outcomes?

2. What is the effectiveness of a synthesized model for applying stress management and biofeedback interven-tions in research utilization?

## 2 METHODS

### 2.1 Conceptual Framework

The study aimed to synthesize a model for applying stress management and biofeedback interventions in research utilization. The conceptual framework was informed by a systematic review and meta-analysis of studies related to stress management and biofeedback interventions. This study was conducted using a seven-stage process, which included: 1) identifying the research questions; 2) conducting a comprehensive literature search; 3) assessing study quality; 4) extracting data; 5) synthesizing the data; 6) analyzing and interpreting the findings; and 7) drawing conclusions and making recommendations. The conceptual framework that was developed from this study included four key components: 1) the target population for the interventions, that is, subjects with mental and physical illnesses; 2) the specific interventions used, that is, electromyogram (EMG), electroencephalogram (EEG), electrocardiography (EKG), heart rate variability (HRV), skin temperature (ST), and skin conductance (SC); 3) the outcomes that were measured, that is, a) the effectiveness of biofeedback interventions in reducing stress and improving health outcomes, and b) the effectiveness of a synthesized model for applying stress management and biofeedback interventions in research utilization; and 4) the contextual factors that may impact the effectiveness of the interventions. The study also identified several potential variables, such as stress, anxiety, and depression, that could be included within each of these components to further refine the conceptual framework. Overall, this study provides crucial insights into the use of stress management and biofeedback interventions in research utilization and offers a useful framework for guiding future research in this area. The conceptual framework is described in Fig. (**1**).

### 2.2 Eligibility Criteria and Search Strategy

The study followed the PRISMA-Protocol guidelines (PRISMA-P) [23]. A systematic review and meta-analysis were conducted in order to identify relevant studies on stress management and biofeedback interventions. Although this research study involves the analysis of existing data from published studies, it is important to note that ethical approval was obtained in order to ensure the protection of the study participants' rights. The specific ethical approval number (reference: RBAC-EC-NUS-1-003/65) was obtained to comply with ethical guidelines and regulatory requirements. All of the data utilized in the analysis were obtained from publicly accessible national and international databases, ensuring transparency and adherence to data protection protocols. Throughout the study, proper attribution will be given to the original authors and studies, acknowledging their contributions and intellectual property rights. The studies were then synthesized in order to develop a proposed model for applying stress management and biofeedback interventions in research utilization. A systematic literature search was conducted in Pubmed, Medline, the Cumulative Index of Nursing and Allied Health Literature (CINAHL), ScienceDirect, and PsycARTICLES online databases for studies published from January, 2017 to May, 2023. The search terms included “stress management,” “biofeedback,” and “research utilization.” A total of 14 studies [24-37] in Table **1** that met the inclusion criteria were identified, and their data were analyzed using a random-effects model meta-analysis. The process was as follows:

#### 2.2.1 Identify the Research Questions

The first step in conducting a meta-analysis is to define the research questions. This should be guided by the purpose and scope of the review. In this study, the questions were: “What is the effectiveness of biofeedback interventions in reducing stress and improving health outcomes?” and “What is the effectiveness of a synthesized model for applying stress management and biofeedback interventions in research utilization?”

#### 2.2.2 Conduct a Comprehensive Literature Search

The next step was to conduct a comprehensive literature search in order to identify relevant studies. This should include using a combination of database-specific keywords and subject headings, and the search should be guided by inclusion and exclusion criteria based on the research questions.

The inclusion criteria for this study were as follows: (1) research design: randomized controlled trials (RCTs) or quasi-experimental research; (2) treatment program: biofeedback interventions alone or biofeedback combined with other techniques; (3) duration of training: interventions with a minimum duration of three weeks; (4) population type: patients, students, women, or workers; and (5) specific clinical symptoms: stress, anxiety, and depression. The exclusion criteria for this study were as follows: (1) studies that were not clinical, *e.g*., descriptive and case studies; (2) those that used non-biofeedback intervention; and (3) those where the sample size was less than twenty.

The search was limited to articles published in English. Following the initial search, duplicates were removed, and the remaining studies were screened based on titles and abstracts. Full-text articles were then assessed for eligibility based on the predefined inclusion and exclusion criteria. The reference lists of selected articles were also reviewed for additional relevant studies. The final set of studies meeting the inclusion criteria were included in the analysis, and their findings were synthesized in order to address the research questions, as shown in Fig. (**2**).

#### 2.2.3 Assess Study Quality

Once the relevant studies were identified, the next step was to assess the quality of each study using a validated quality assessment tool, such as the Critical Appraisal Skills Programme (CASP) tool [38]. This step is essential in order to ensure that studies are of sufficient quality and that the findings are reliable.

#### 2.2.4 Data Extraction

Subsequently, a standardized data extraction form was utilized to extract pertinent information from each study. This encompassed various details, including the publication year, research design, treatment program, training duration, population type, sample size, and specific clinical symptoms. These particulars are presented in Table **1**.

#### 2.2.5 Synthesize the Data

The data collected from each study could then be synthesized using statistical methods in order to estimate overall effect sizes, such as mean differences or odds ratios. This involved pooling the data from individual studies to provide a summary estimate of the effect of biofeedback interventions on stress reduction and health outcomes.

#### 2.2.6 Analyze and Interpret the Findings

Once the data had been synthesized, the next step was to analyze and interpret the findings. This involved examining the overall effect size and any variations between studies and subgroups. It was also essential to assess the heterogeneity of the studies and the potential sources of bias in the data.

When conducting a systematic review and meta-analysis on stress management and biofeedback interventions, it is important to assess the robustness of the synthesized results and the risk of bias due to missing results. Two common methods used for this study were sensitivity analyses and assessment of reporting bias.

1) Sensitivity analyses were conducted in order to examine the robustness of the synthesized results by testing the impact of various methodological choices or assumptions on the overall findings. These analyses can include excluding studies with a high risk of bias, exploring the influence of different statistical models or effect size calculations with a random-effect meta-analysis. Statistical analysis was performed using SPSS version 29. Funnel plots were used for investigating publication bias, and the scatterplots of the treatment effects estimated from individual studies increased as the sample size increased. Heterogeneity was examined using I2 and Q statistics, and the difference in the impact of specific sub-groups of studies was also assessed. By conducting sensitivity analyses, researchers can evaluate whether the results of the meta-analysis are consistent and reliable across different scenarios, thereby enhancing confidence in the findings.

2) Analyzing the reporting of bias, such as selective outcome bias, can arise when studies with positive or statistically significant results are more likely to be published or reported compared to studies with negative or non-significant results. These biases can distort the overall findings of a meta-analysis. In order to assess the risk of bias due to missing results, researchers can employ various methods, including funnel plots, as described in Fig. (**3**). These techniques help to visualize and quantify the potential impact of the biases on the synthesized results, allowing for a more comprehensive evaluation of the evidence.

By conducting sensitivity analyses and assessing reporting biases, researchers can enhance the robustness and reliability of the synthesized results in the systematic review and meta-analysis of stress management and biofeedback interventions. These methods provide insights into the potential impact of methodological choices, assumptions, or biases on the overall findings and help researchers draw more valid conclusions from the synthesized evidence.

#### 2.2.7 Draw Conclusions and Make Recommendations

Finally, the results of the meta-analysis can be used to draw conclusions and to make recommendations. For example, the meta-analysis can identify the most effective types of biofeedback interventions or the populations for which such interventions are most effective. These conclusions and recommendations can also help to guide future research in the clinical nursing field.

In addition to the above, the application of sample selection criteria based on medical subject headings further strengthens the rigor of the process. By using standardized and recognized subject headings, the inclusion of relevant studies is enhanced, minimizing the risk of omitting important research. This systematic approach ensures that studies included in the analysis are aligned with the specific topics of interest, such as stress management and biofeedback interventions.

The incorporation of diverse populations, intervention types, and outcome measures in the synthesized model also contributes to its robustness. By considering studies with varied characteristics, the model captures a broader spectrum of the effectiveness of stress management and biofeedback interventions. This approach acknowledges the heterogeneity that exists within the field and allows for a more comprehensive understanding of how these interventions may impact different populations and under different circumstances.

Through the integration of multiple studies that meet the specified criteria, the synthesized model provides a comprehensive summary of the available evidence. This approach allows for the identification of common themes, trends, and patterns across the selected studies, strengthening the overall findings. By carefully analyzing and synthesizing the collective results, the model offers valuable insights into the effectiveness of stress management and biofeedback interventions, informing future research and practice in the field.

Overall, the combination of rigorous sample selection criteria, including medical subject headings, and the incorporation of diverse studies enhances the credibility and reliability of the proposed model. It provides a robust foundation for advancing knowledge and understanding in the areas of stress management and biofeedback interventions, facilitating evidence-based decision-making and guiding the integration of these interventions into healthcare practices.

## 3 RESULTS

The encompassed various details, including the publication year, research design, treatment program, training duration, population type, sample size, and specific clinical symptoms are mentioned in Table **1**.

### 3.1 Meta-analysis of the Biofeedback Interventions

This study provides insights into the effectiveness and outcomes of using synthesized models through a comparative study after being synthesized and then divided into the following categories: 1) applying biofeedback interventions alone or biofeedback interventions together with relaxation, mindfulness, or psycho-education techniques to reduce stress, anxiety, and depression; 2) combining biofeedback interventions with relaxation, mindfulness, psycho-education techniques, or biofeedback interventions alone; 3) comparison of the effect size among subgroups, such as treatment programs, duration of training, and population type. Details are as follows:

The funnel plot for investigating the publication bias in this study showed that the effect sizes were based on the increasing estimation, which depended on the increase of the sample size of studies. The scatter plots of the treatment effects estimated from individual studies, explaining the association of publication probability with the statistical significance of previous study results, showed absent bias, as shown in Fig. (**3**).

The results of the forest plot analysis found that the I^2^ test showed substantial heterogeneity, indicating that statistical heterogeneity was typically considered and corroborated the inference of the Cochran Q test that statistical heterogeneity existed (Q = 63.59, df = 23, *p* < .001). The effect sizes of the studies are shown in Fig. (**4**).

The test of the difference among subgroups in each variable showed that only population types are different, and also, the effect size of the treatment of patients was significantly stronger than for the workers. On the other hand, the statistical test showed that treatment type and duration of training were not different among subgroups, as mentioned in Table **2**.

The study results are shown above; however, the possible causes of heterogeneity among the study results in the synthesized model for applying stress management and biofeedback interventions in research utilization can be explained in terms of subgroup analysis and meta-regression as follows:

1. Subgroup analysis involves dividing the studies into different subgroups based on specific characteristics or variables in order to investigate whether the treatment effects of stress management and biofeedback inter-ventions differ across these subgroups. For example, potential subgroups could be population type, the type of biofeedback interventions, the duration of treatments, or three categorized symptoms. By comparing the treatment effects within each subgroup, it is possible to identify the potential factors contributing to the heterogeneity in the study results.

2. Meta-regression is a statistical technique that allows for the exploration of the relationship among study-level characteristics (such as research design, treatment program, the duration of training, dependent variables, population type, sample size, publication year from 2017 to 2023, and other study qualities). Through meta-regression analysis, it is possible to quantify the impact of these covariates on the heterogeneity of the results and to identify potential sources of variation. This method helps to determine whether certain study characteristics are significantly associated with the treatment effects, thus explaining the heterogeneity observed.

The utilization of subgroup analysis and meta-regression in systematic reviews and meta-analyses allows for a deeper exploration of the potential sources of heterogeneity among study results. These analytical techniques are valuable in identifying factors that may contribute to variations in treatment effects, shedding light on the effectiveness of stress management and biofeedback interventions within specific subgroups or under specific conditions. This knowledge is crucial in refining interventions, identifying target populations, and informing future research directions.

In this study, subgroup analyses were conducted to examine the differences in treatment effects among various variables. The results revealed that the only significant difference observed was in population types. Specifically, the effect size of stress management and biofeedback interventions was found to be significantly stronger for patients compared to workers. This finding suggests that these interventions may be particularly beneficial for individuals dealing with health-related challenges, as they demonstrated more pronounced improvements compared to those in occupational settings.

The differential effectiveness of stress management and biofeedback interventions across populations has important clinical implications. Healthcare providers and practitioners can use this information to tailor interventions to specific groups, ensuring that patients receive the most appropriate and effective treatments based on their unique needs and circumstances. For instance, healthcare professionals working with patients may consider integrating stress management and biofeedback interventions as part of their treatment plans to optimize outcomes and enhance patient well-being.

Furthermore, these findings highlight the importance of further investigating the specific factors that contribute to the differential treatment effects between patient and worker populations. Future research endeavors could delve deeper into understanding the underlying mechanisms and contextual factors that may influence the effectiveness of stress management and biofeedback interventions in these distinct settings. This knowledge can inform the development of targeted interventions tailored to the specific needs of different populations, ultimately improving the delivery and impact of stress management strategies in clinical practice.

## 4 DISCUSSION

Stress, anxiety, and depression are distinct psychological conditions, but they often coexist and share certain similarities in terms of symptoms and underlying mechanisms. In other words, biofeedback interventions can have similar effects on the autonomic nervous system (ANS) [39, 40, 18] and the adrenal glands in terms of both anxiety and depression due to the interconnectedness of these systems and the physiological responses associated with stress [41, 42]. Biofeedback interventions aim to modulate the ANS response by providing real-time feedback on physiological parameters [43, 44]. Individuals can learn to consciously regulate their bodily functions by observing the feedback and making adjustments, the same mechanism of biofeedback interventions in the autonomic nervous system (ANS) and adrenal glands that responds to the physiological mechanism in those with anxiety, depression, and stress [45-48].

1) The ANS is responsible for regulating involuntary bodily functions, including heart rate, blood pressure, respiration, and digestion. It consists of two major branches: the sympathetic nervous system (SNS) and the parasympathetic nervous system (PNS). In anxiety, the SNS is often overly activated, resulting in the “fight-or-flight” response. This leads to increased heart rate, rapid breathing, elevated blood pressure, and heightened muscle tension. In depression, there is often an imbalance between the SNS and PNS, with reduced activity in the PNS. This can result in a decreased heart rate, lowered blood pressure, and reduced physiological arousal [49, 50].

2) Adrenal gland response: The adrenal glands, located on top of the kidneys, produce hormones that are involved in the body's stress response. The two main hormones released by the adrenal glands are cortisol and adrenaline (epinephrine). During anxiety, the adrenal glands may release excessive amounts of adrenaline, contributing to heightened arousal and anxiety symptoms [51], and during depression, there may be dysregulation in the cortisol response. Some individuals with depression exhibit elevated cortisol levels, particularly in chronic or severe cases. Biofeedback interventions can indirectly influence the adrenal gland response by helping individuals manage their stress, anxiety, and depression and regulate their physiological arousal [52-54].

It is important to note that while biofeedback interventions show promise in terms of managing stress, anxiety, and depression, they are typically used as part of a comprehensive treatment plan that may include other relaxation techniques, psychotherapy, and lifestyle modifications. For instance, by learning relaxation techniques and stress reduction strategies, individuals can potentially modulate the release of cortisol and adrenaline, leading to a more balanced stress response [55-59].

When it comes to the effectiveness of biofeedback interventions for stress, anxiety, and depression, the specific duration of training can vary depending on several factors, including the individual's condition, the specific biofeedback modality used, and the intensity and frequency of training sessions [60-63]. Therefore, the response to biofeedback interventions can be influenced by various individual factors, making it challenging to determine a specific time period for training. Below is an explanation of why the length of time for training may not make a significant difference.

1. Individual variability: People differ in their response to interventions, including biofeedback training. Some individuals may show improvement in symptoms relatively quickly, while others may require more time. The response to biofeedback can depend on various factors, such as the severity of symptoms, individual differences in learning and self-regulation abilities, and overall treatment adherence [64-69].

2. Learning curve: Biofeedback involves learning to regulate physiological responses by receiving real-time feedback. Initially, individuals may need time to understand the feedback and to develop the skills necessary for self-regulation [70-73]. However, once they grasp the techniques and become proficient in applying them, the benefits may be observed more consistently.

3. Treatment context: The duration of biofeedback training can also depend on the treatment context and the specific goals being addressed. Some individuals may receive biofeedback as a standalone treatment, while others may use it as part of a comprehensive treatment plan that includes therapy, medication, or other interventions [74-76]. The overall treatment duration and goals will influence the length of biofeedback training.

4. Maintenance and generalization: After initial training, individuals may continue to practice biofeedback techniques independently to maintain the benefits achieved. The duration of training can also depend on the extent to which individuals incorporate these techniques into their daily lives and continue to use them beyond the formal training period [77].

It can be concluded that the specific duration of biofeedback training for stress, anxiety, and depression can vary based on individual needs and treatment goals. The effectiveness of biofeedback is often determined by such factors as regular practice, personalized guidance, and ongoing support from a qualified healthcare professional or therapist. While research studies may provide insights into the effectiveness of biofeedback interventions, the optimal duration of training is still an area of ongoing investigation and may vary depending on individual circumstances [78-80].

Biofeedback interventions have been shown to be effective in managing stress, anxiety, and depression in both patients and normal individuals, such as students, women, and workers, as described in this study. However, there are certain differences among those groups in terms of their responses to these interventions. Here are some specific explanations for these differences.

1. Psychophysiological awareness: Patients with stress, anxiety, or depression often have limited awareness of their physiological responses to stressors. Biofeedback interventions can help increase their psychophysiological awareness by providing real-time feedback concerning their physiological parameters, such as heart rate, skin conductance, or muscle tension. This increased awareness enables patients to recognize and regulate their physiological responses, leading to reduced stress and anxiety levels. Compared with normal individuals, biofeedback interventions can target underlying psychological processes, such as emotional regulation, cognitive restructuring, and relaxation, which can be more relevant to healthy individuals [81-83].

2. Self-regulation skills: Individuals without clinical diagnoses often possess well-developed self-regulation skills, allowing them to manage stress, anxiety, and depression more effectively. Biofeedback interventions can further enhance these skills by providing individuals with additional tools and techniques to modulate their physiological responses. Consequently, individuals may exhibit a quicker and more efficient response to biofeedback interventions compared to patients [16, 84].

3. Underlying pathology: Patients with clinical diagnoses of stress, anxiety, or depression may have underlying physiological or psychological pathologies that contribute to their symptoms. While biofeedback interventions can be beneficial for these individuals, additional therapeutic approaches may be necessary in order to address the underlying causes of their conditions. Individuals, on the other hand, may respond well to biofeedback interventions alone, as their symptoms may be primarily related to situational stressors rather than underlying pathologies [5].

Biofeedback interventions or interventions, together with other relaxation techniques, can be helpful in managing these conditions by providing individuals with real-time information about their physiological responses, allowing them to learn self-regulation techniques. While there is limited research directly comparing the effects of biofeedback interventions on stress, anxiety, and depression, this study provides some interesting information on their potential responses to biofeedback interventions alone and the interventions combined with other relaxation techniques that can be described as follows: 1) Stress is a natural response to challenging or threatening situations. However, chronic or excessive stress can have negative effects on mental and physical health. Studies have demonstrated that biofeedback training alone targeting heart rate variability (HRV), a measure of autonomic nervous system activity, can lead to decreased stress levels and increased resilience to stressors [85-87]. Biofeedback interventions combined with diaphragmatic breathing, progressive muscle relaxation, and mindfulness meditation have also been used to alleviate stress [88]. 2) Anxiety disorders involve excessive and persistent worry, fear, and unease. Biofeedback training has been explored as a complementary approach to treating anxiety. Several studies have demonstrated the efficacy of biofeedback interventions in reducing anxiety symptoms. For instance, biofeedback training alone targeting skin conductance, a measure of sympathetic nervous system arousal, has been shown to reduce anxiety in individuals with generalized anxiety disorder [82]. Additionally, biofeedback techniques, such as heart rate variability biofeedback and electroencephalographic (EEG) biofeedback, have shown promise in alleviating symptoms of anxiety [55, 88]. 3) Depression is a mood disorder characterized by persistent feelings of sadness, loss of interest, and impaired functioning. Biofeedback training programs have been investigated as adjunctive treatments for depression. Although more research is needed, some studies have suggested that biofeedback techniques, such as HRV biofeedback and EEG biofeedback, may have the potential to reduce depressive symptoms [17, 89, 90]. These interventions aim to regulate physiological processes associated with depression, such as autonomic nervous system dysregulation and abnormal brainwave patterns. This study investigated the effects of heart rate variability biofeedback on depression symptoms. The results showed a significant reduction in depressive symptoms following the HRV biofeedback interventions.

Biofeedback interventions for stress, anxiety, and depression often utilize smaller-scale devices due to several advantages that they offer over comprehensive large-scale devices [70]. Here are specific explanations outlining these advantages: smaller-scale biofeedback devices are often portable and can be easily used in various settings, such as homes, clinics, or workplaces. Their compact size allows individuals to carry them and use them whenever needed, promoting accessibility and convenience [91]. When compared to larger-scale devices, smaller biofeedback devices tend to be more affordable, and this cost-effectiveness makes them more accessible to individuals seeking self-help or non-clinical interventions for stress, anxiety, or depression [92]. Smaller-scale biofeedback devices often have user-friendly interfaces and intuitive designs, which enhance user engagement. These devices typically provide real-time feedback on physiological parameters, such as heart rate, skin conductance, or breathing patterns. By receiving immediate feedback, individuals can learn to self-regulate their physiological responses, empowering them to actively manage their stress, anxiety, or depression [93]. Moreover, smaller-scale biofeedback devices often offer customizable features that allow individuals to tailor interventions to their specific needs. They may provide options for adjusting parameters, setting goals, or selecting preferred relaxation techniques. This personalization promotes individualized treatment approaches, which can be crucial in managing stress, anxiety, or depression [93].

In terms of large-scale comprehensive devices, such as electromyography (EMG) biofeedback training [26, 62], EMG utilizes electronic devices to measure and provide feedback on muscle activity. It is less commonly applied than other types of biofeedback due to its limited applications and the equipment complexity it involves. Compared to other forms of biofeedback, such as HRV biofeedback or ST and SC biofeedback, these forms have broader applications and can address a wider range of conditions. In addition, effective implementation of EMG biofeedback training requires specialized training and expertise. Professionals need to be skilled in interpreting and analyzing electromyographic data in order to provide appropriate feedback and guide the therapeutic process. This level of expertise may not be readily available in all clinical settings, which can limit the widespread use of EMG biofeedback training. The equipment used in EMG biofeedback training can be costly, and not all healthcare facilities or individuals may have access to it. Additionally, ongoing maintenance and calibration of the equipment are necessary to ensure accurate measurements and reliable feedback [71-73].

In summary, while EMG biofeedback training has demonstrated effectiveness in certain cases, its limited applications, equipment complexity, training expertise requirements, and cost and accessibility factors contribute to its relatively lower frequency of use compared to other types of biofeedback.

It is important to note that the application of biofeedback techniques, including EMG biofeedback training, can vary across different clinical settings and individual cases. The reasons mentioned above are general factors that may contribute to the relatively lower utilization of EMG biofeedback training compared to other types of biofeedback, but individual circumstances and clinical expertise should guide the selection of the most appropriate biofeedback modality. The factors mentioned above provide a general perspective on why EMG biofeedback training may be applied less frequently than other forms of biofeedback. However, the choice of biofeedback modality should always be based on individual needs, clinical judgment, and the available evidence.

### 4.1 Implications and Limitations of the Study

#### 4.1.1 Implications

The implications of the results for practice, policy, and future research for the synthesized model of applying stress management and biofeedback interventions in research utilization, based on a systematic review and meta-analysis, are as follows:

1) Practice implications: The synthesized model derived from the systematic review and meta-analysis provides valuable insights for practitioners involved in stress, anxiety, and depression interventions. The results suggest that biofeedback interventions alone or combined with other relaxation techniques can be beneficial in improving health outcomes. Practitioners can consider integrating these techniques into their existing stress, anxiety, and depression management protocols, as these techniques can enhance self-regulation skills, providing individuals with real-time feedback on their physiological responses and facilitating effective stress, anxiety, and depressive reduction. Implementing this synthesized model in practice can lead to improved health outcomes in terms of stress, anxiety, and depression and essentially enhance the overall effectiveness of stress management interventions.

2) Policy implications: The findings from the systematic review and meta-analysis have important implications for policy development in the field of stress, anxiety, and depression management. Policymakers and healthcare organizations can consider incorporating biofeedback interventions into existing management guidelines and protocols. Recognizing the effectiveness of biofeedback techniques for stress, anxiety, and depression reduction, policies can be developed to promote the integration of biofeedback training within healthcare systems. This can include providing training opportunities for healthcare professionals to acquire the necessary skills and knowledge to deliver biofeedback interventions effectively. By incorporating biofeedback training into policy frameworks, policymakers can facilitate the widespread adoption of evidence-based stress, anxiety, and depression management practices across various healthcare settings.

3) Future research implications: The synthesized model derived from this systematic review and meta-analysis also highlight avenues for future research, typically in terms of stress management and biofeedback interventions. Further studies can explore the long-term effects of biofeedback interventions on stress management outcomes, and research can focus on identifying the specific mechanisms through which biofeedback techniques exert their effects, shedding light on the underlying physiological and psychological processes involved. In addition, future research can investigate the optimal duration, frequency, and delivery methods of biofeedback interventions in order to maximize their effectiveness. Comparative studies examining the effectiveness of different biofeedback modalities, specifically between smaller-scale devices and comprehensive professional large-scale devices, or their combination with other therapeutic approaches, can also provide valuable insights. By addressing these research gaps, future studies can contribute to the refinement and advancement of stress management practices incorporating biofeedback interventions.

These implications highlight the potential for integrating biofeedback interventions into stress, anxiety, and depression management approaches, informing practice, policy, and future research directions in this field.

#### 4.1.2 Limitations

The limitations of the evidence included in the review and the review processes used in the synthesized model for applying stress management and biofeedback interventions in research utilization, based on a systematic review and meta-analysis, are as follows.

1) Publication bias: The biofeedback evidence included in the review may be subject to publication bias, where studies with statistically significant results are more likely to be published than those with non-significant or negative results. This bias can lead to an overestimation of the effectiveness of biofeedback interventions if studies with positive outcomes are disproportionately represented in the review. Efforts to identify and include unpublished studies or gray literature can help mitigate this bias, but it may still exist to some extent.

2) Study design: The evidence included in the review may be limited by the types of study designs available. Randomized controlled trials (RCTs) are considered the gold standard for evaluating intervention effectiveness. However, if there is a lack of sufficient RCTs in the field of stress, anxiety, and depression management together with biofeedback interventions, the review may have to include other study designs, such as quasi-experimental studies. These study designs may have inherent limitations in terms of controlling for confounding variables, establishing causality, or minimizing bias. Therefore, the overall strength of the evidence may be compromised due to the inclusion criteria of the study.

3) Heterogeneity: Another limitation is the potential heterogeneity among the included studies. The biofeedback studies included in the systematic review and meta-analysis may vary in terms of participant characteristics, such as patients and normal individuals, intervention protocols, or the outcome measures used. This heterogeneity can affect the comparability and generalizability of the results. Although statistical methods, such as subgroup analyses and sensitivity analyses, can be employed to explore and address heterogeneity, it may not be possible to fully account for all sources of heterogeneity in the synthesized model. Therefore, the generalizability of the findings may be limited to the specific population, intervention protocols, and outcome measures included in the analyzed studies.

4) Variation in intervention protocols: The evidence included in the review may encompass a wide range of intervention protocols, which can introduce heterogeneity and limit comparability. Biofeedback interventions can vary in terms of the specific techniques used, the duration of training, the frequency and duration of sessions, and the expertise and qualifications of the providers. This variation in intervention protocols may make it challenging to draw definitive conclusions about the effectiveness of specific biofeedback techniques or to determine the optimal parameters for implementation. It is important to consider this variation when interpreting the review findings and applying them to practice.

5) Outcome measures: The evidence included in the review may rely on subjective self-report measures of stress, anxiety, and depression, which can be influenced by various factors, including social desirability bias or recall bias. While self-report measures are commonly used in stress management research, they may lack objectivity and precision. Including objective physiological measures, such as heart rate variability or cortisol levels, alongside self-report measures can strengthen the evidence base and provide a more comprehensive understanding of the effects of biofeedback interventions.

In summary, while the synthesized model provides valuable insights into stress management and biofeedback interventions, it is important to acknowledge these limitations in interpreting and applying the findings appropriately.

## CONCLUSION

In conclusion, while the results of this synthesized model support the effectiveness of stress management and biofeedback interventions in reducing stress levels and improving health outcomes, it is important to consider certain limitations. One such limitation is the small sample size of the studies included in this analysis. The small sample size may impact the generalizability of the findings and limit the statistical power to detect significant effects. Therefore, caution should be exercised when interpreting the results, and future research with larger sample sizes is warranted to strengthen the evidence base. Additionally, the wide heterogeneity of subjects and interventions across the studies included in this analysis is another aspect to consider. The diverse range of subjects, including variations underlying health conditions, as well as the variability in the types and durations of interventions, may introduce potential confounding factors and limit the ability to draw definitive conclusions. Future studies should aim for more consistent subject selection criteria and standardized interventions to minimize heterogeneity and enhance comparability between studies.

Despite these limitations, the proposed model for applying stress management and biofeedback interventions in research utilization can still serve as a valuable tool for enhancing knowledge and skills in these areas. By synthesizing the available evidence, it provides a foundation for understanding the potential benefits of these interventions and guiding their implementation in various healthcare settings. However, further research is necessary to evaluate the long-term effects of stress management and biofeedback interventions and to explore their integration into broader healthcare practices. Such research endeavors will help address the existing limitations and provide a more comprehensive understanding of the impact of these interventions on stress reduction and overall health outcomes.

## RECOMMENDATIONS

Based on the findings of our study, several recommendations can be made to guide the implementation of stress management and biofeedback interventions in clinical practice. Firstly, healthcare professionals should consider tailoring these interventions to specific populations, taking into account the differential treatment effects observed. For patients, stress management and biofeedback interventions can be integrated as effective adjunctive therapies to address stress-related challenges and improve overall well-being [72]. Specific techniques, such as deep breathing exercises, progressive muscle relaxation, and heart rate variability biofeedback, have shown promise in patient populations [91]. On the other hand, for workers, strategies that focus on stress reduction in occupational settings, such as workplace mindfulness programs or resilience-building workshops, may be particularly relevant [92].

Secondly, our findings highlight the importance of addressing population-specific factors in the delivery of stress management interventions. Healthcare professionals should consider individual characteristics, such as health status, work demands, and environmental factors, when designing and implementing these interventions. For example, in patients with chronic conditions, stress management interventions can be integrated into disease management programs to improve symptom management and overall quality of life [92]. In occupational settings, interventions that target work-related stressors and promote work-life balance can be instrumental in reducing stress and enhancing worker well-being [93].

Lastly, further research is warranted to explore the underlying mechanisms and contextual factors that contribute to the differential treatment effects observed. Future studies could investigate the specific patient characteristics, occupational factors, or intervention components that influence the effectiveness of stress management and biofeedback interventions. This knowledge can inform the development of personalized and evidence-based approaches to stress management in diverse populations.

In conclusion, our study's findings suggest that stress management and biofeedback interventions have varying effects across different populations. Tailoring interventions to specific populations and considering population-specific factors in their implementation can optimize outcomes and promote individualized care. By recognizing these variations and incorporating evidence-based strategies, healthcare professionals can effectively integrate stress management and biofeedback interventions into clinical practice, ultimately improving patient outcomes and well-being.

It is also important to note that the synthesized model and recommendations provided in this study serve as a starting point for guiding the application of stress management and biofeedback interventions in clinical practice. As the field continues to evolve and new evidence emerges, ongoing research and collaboration between researchers, clinicians, and educators will be crucial in refining and expanding our understanding of the optimal utilization of biofeedback interventions in various clinical contexts.

## Figures and Tables

**Fig. (1):**
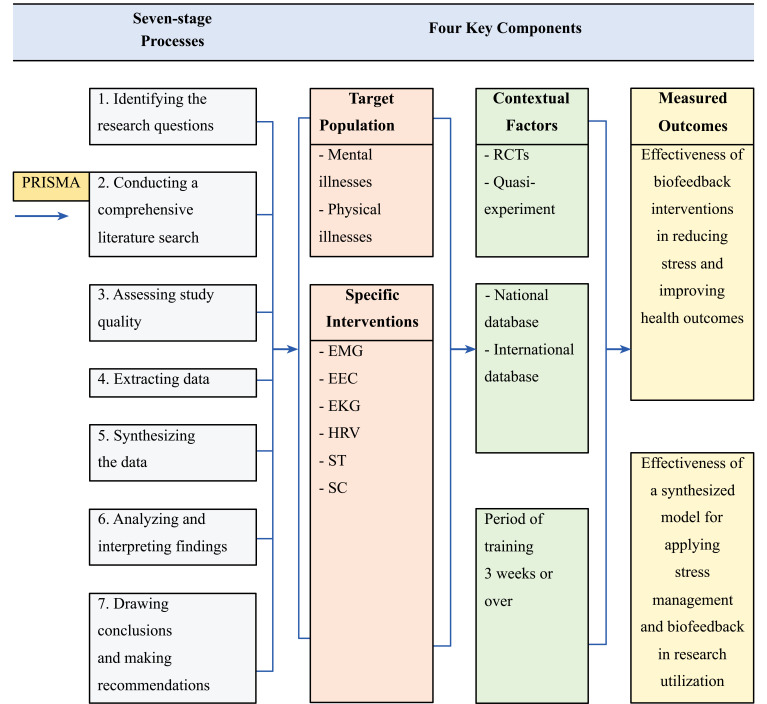
Conceptual framework of a synthesized model for applying stress management and biofeedback interventions in research utilization.

**Fig. (2):**
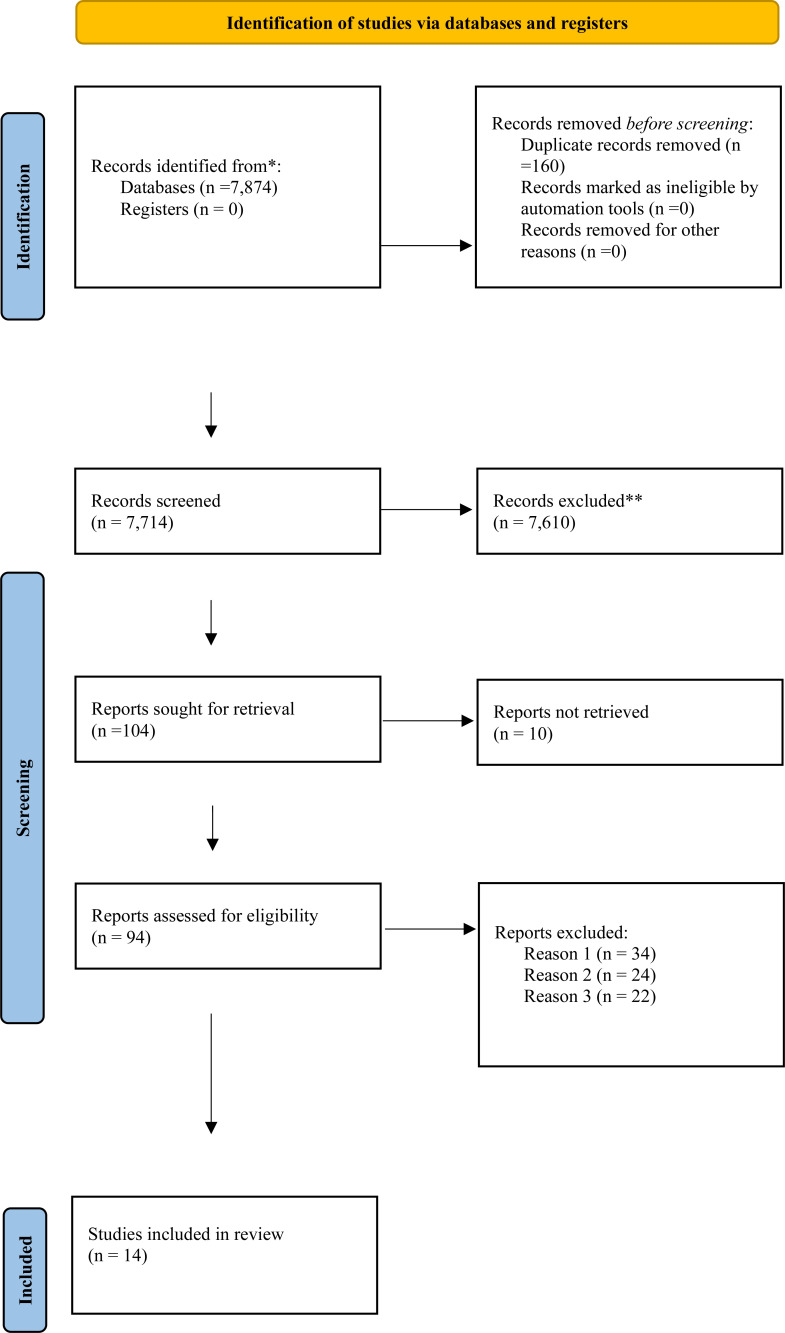
PRISMA flow diagram adapted to examine included studies.

**Fig. (3):**
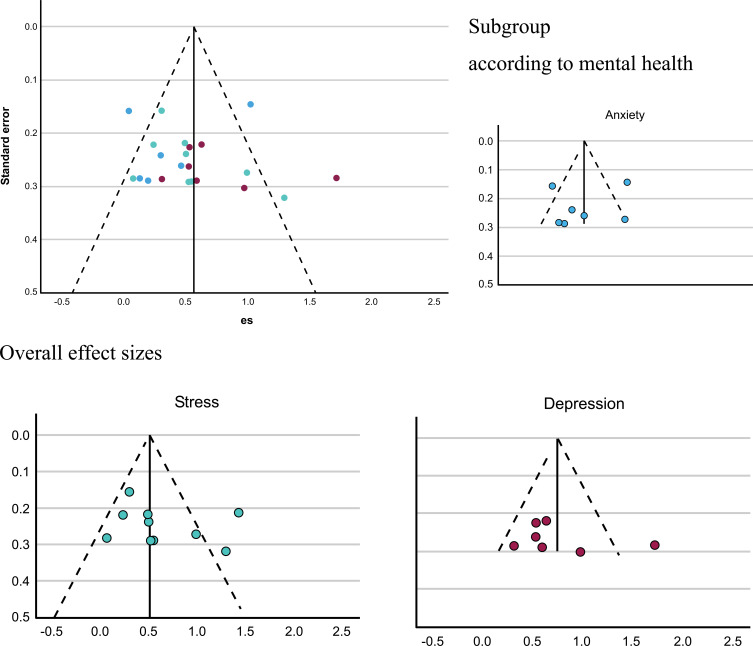
Funnel plot of the effect size in each study.

**Fig. (4):**
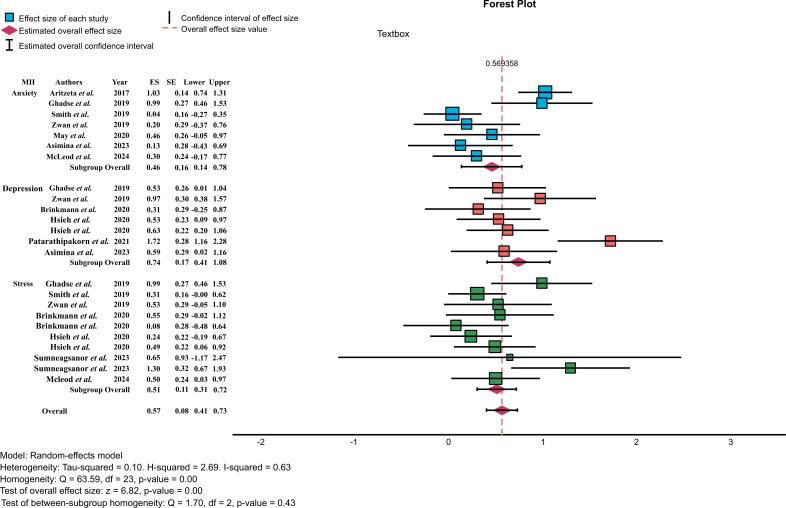
Forest plot of effect size.

**Table 1:** The relationship among the study-level characteristics of the included studies.

**Author** **and Year/Refs**	**Research Design**	**Treatment Program**	**Duration of Training**	**Population** **Type**	**Sample** **Size**	**Specific Clinical Symptoms**
McLeod *et al*. 2021 [24] Canada	Quasi-experimental	Breathing relaxation with Biofeedback	3 weeks	Adolescent	87	Anxiety and stress
Patarathipakorn *et al*. 2021 [25] Thailand	RCT	Meditation with a Biofeedback Program	6 weeks	Diabetes patients with mild depression	34	Stress and depression
Lazaridou *et al*. 2023 [26] U.S.A.	RCT	EMG Biofeedback	8 weeks	Chronic low back pain	27	Anxiety and depression
Sumneangsanor *et al*.2022 [27] Thailand	RCT	Biofeedback and music therapy	6 weeks	Thai patients living with cancer receiving palliative care	44	Stress
Ghadse et. Al. 2019 [28] India	RCT	Biofeedback- assisted relaxation technique	4 weeks	Patients with substance abuse disorders	60	Depression Anxiety Stress
Laudenslager *et al*. 2019 [29] U.S.A.	RCT	Psychoeducation Paced Respiration and Relaxation	8 weeks	Caregivers and patients with bone marrow transplant	155	Distress Depression Anxiety
Brinkmann et.al. 2020 [30] Germany	RCT	1.HRV-Biofeedback 2. Mindfulness	6 weeks	Healthy adults in work context	50	TICS-SSCS chronic stress Depression
Hsieh *et al*., 2020 [31] Taiwan	RCT	1. Biofeedback training (BT) 2. Smartphone- delivered BT (SDBT)	6 weeks	Psychiatric ward nurses from three hospitals with workplace violence	88	Depression Stress
Smith *et al*. 2019 [32] U.S.A.	RCT	Brief mindfulness-based training and biofeedback in smartphone application	4 weeks	Employee in workplace	169	Stress Anxiety Negative effects
Mousavi *et al*., 2019 [33] Iran	Quasi-experimental study	Acceptance and Commitment Therapy and Biofeedback	8 weeks	Women with chronic psychosomatic experienced lower back pain	20	Psychological health
Zwan *et al*. 2019 [34] Netherlands	RCT	HRV Biofeedback training with psycho-education	5 weeks	Pregnant and non-pregnant who suffered from stress	50	Depression Anxiety Stress
Hoseinpourfard *et al*. 2020 [35] Iran	RCT	HRV Biofeedback with abdominal breathing *via* chest breathing	4 weeks	Patients with sleep disorders	48	Pittsburgh Sleep Quality Index
May *et al*. 2020 [36] U.S.A.	Quasi-experimental study	HRV Coherence Biofeedback (HRVCB) Training with high-intensity interval training	4 weeks	College students	60	Academic success
Aritzeta *et al*. 2017 [37] Spain	RCT	Deep breathing with guided imagery and muscle relaxation	8 weeks	Psychology undergraduates	152	State Anxiety

**Table 2:** Comparison of the effect size among subgroups.

**Variables**	**Effect Size**						
	**n**	**Min**	**Max**	**Mean + SD**	**Median**	**Q (df)**	***p***
**Treatment type**	-	-	-	-	-	4.02 (3)	0.26
Biofeedback	7	0.13	0.65	0.47+0.20	0.53	-	-
Biofeedback with relaxation	8	0.30	1.30	0.76+0.36	0.76	-	-
Biofeedback with mindfulness	6	0.04	1.72	0.50+0.62	0.31	-	-
Biofeedback with psychoeducation	3	0.20	0.98	0.57+0.39	0.53	-	-
**Duration of Training**	-	-	-	-	-	0.68 (4)	0.95
3 weeks	2	0.30	0.50	0.40+0.14	0.40	-	-
4 weeks	6	0.04	1.00	0.55+0.38	0.49	-	-
5 weeks	3	0.20	0.98	0.57+0.39	0.53	-	-
6 weeks	9	0.08	1.30	0.53+0.35	0.53	-	-
8 weeks	2	0.13	0.59	0.36+0.33	0.36	-	-
**Participants**	-	-	-	-	-	8.49 (3)	0.04
Patients	8	0.13	1.72	0.86+0.50	0.82	patients > workers	0.01
Students	4	0.30	1.03	0.57+0.32	0.48	-	-
Women	3	0.20	0.98	0.57+0.39	0.53	-	-
Workers	9	0.04	0.63	0.35+0.21	0.31	-	-

**Note: **Significant level at .05.

## Data Availability

The data that support the findings of this study are available from the corresponding author [S.V] upon reasonable request.

## STANDARDS OF REPORTING

PRISMA-P guidelines and methodology were followed.

## SUPPLEMENTARY MATERIAL

PRISMA-P checklist is available as supplementary material on the publisher’s website along with the published article. Supplementary material is available on the publisher’s website along with the published article.
